# Long-term daily feeding of cannabidiol is well-tolerated by healthy dogs

**DOI:** 10.3389/fvets.2022.977457

**Published:** 2022-09-21

**Authors:** Sophie Bradley, Scott Young, Anne Marie Bakke, Lucy Holcombe, Daniel Waller, Alysia Hunt, Kathleen Pinfold, Phillip Watson, Darren W. Logan

**Affiliations:** Waltham Petcare Science Institute, Waltham-on-the-Wolds, Melton Mowbray, United Kingdom

**Keywords:** food safety, pet food ingredients, cannabinoids, canine, dog, CBD, cannabidiol

## Abstract

Cannabidiol (CBD) containing dog food and treats are widely commercially available, mirroring the growing popularity of CBD as a supplement for humans. Despite this, experimental evidence of the safety and efficacy of long-term oral exposure in dogs is lacking. The purpose of this study was to address the gap in knowledge around the longer-term suitability and tolerance of a broad-spectrum CBD (THC-free) distillate in clinically healthy dogs. The study was a randomized, placebo-controlled, and blinded study where one group of twenty dogs received daily CBD capsules at a dose of 4 mg/kg of body weight (BW) for a period of 6 months. The control group of twenty dogs received placebo capsules. A comprehensive suite of physiological health measures was performed throughout the study at baseline, and after 2, 4, 10, 18, and 26 weeks of exposure, followed by 4 weeks of washout. CBD concentrations were measured at the same cadence in plasma, feces and urine. Health measures included biochemistry, hematology, urinalysis, in addition to fortnightly veterinary examinations, twice daily well-being observations, and a daily quality-of-life survey. Biochemistry and hematology showed no clinically significant alterations apart from a transient elevation in alkaline phosphatase (ALP) in just over half of the dogs receiving CBD. This elevation was observed in the absence of concurrent elevations of other liver parameters, and without any adverse effects on health and wellbeing. Furthermore, bone alkaline phosphatase (BALP) was simultaneously elevated with a significant, strong (*r* > 0.9) positive correlation between the two measures, suggesting that the elevation of total ALP was at least partly due to the bone-derived isoform. This study provides evidence that a once-daily oral dose of 4 mg CBD/kg BW is well tolerated in clinically healthy dogs for a duration of 6-months.

## Introduction

*Cannabis sativa/C. indica* is an herbaceous plant of Asian origin that has been cultivated for centuries. Different parts of the plant have been utilized across diverse applications including for fiber, fuel, nutrition and medicine (1). It is thought to be the oldest cultivated plant (2, 3) and its extensive list of bioactive compounds is documented to be unrivaled when compared to other botanicals (4). Specifically, research to date has identified it contains over 120 phytocannabinoids alongside at least 445 other phytochemicals, including terpenoids, flavonoids, and sterols (5). Tetrahydrocannabinolic acid (THCA) and cannabidiolic acid (CBDA) are the two primary phytocannabinoids produced by the plant and are found at different concentrations dependent upon the species and strain. These natural products are decarboxylated through the process of cooking, heating or drying to form tetrahydrocannabinol (THC), a psychoactive cannabinoid, and cannabidiol (CBD), a non-psychoactive cannabinoid. Cannabis cultivar products are commonly differentiated into either marijuana or hemp. Cultivars that contain appreciable levels of THCA are typically used to produce THC-rich marijuana. Conversely, hemp products are from plants characterized as having low levels of THCA but tend to have high levels of CBDA.

CBD has attracted an abundance of interest over recent years due to proposed anti-oxidative, anti-inflammatory and anti-necrotic effects, making it a potential candidate for therapeutic management of ailments and conditions such as Alzheimer's disease (6, 7), Parkinson's disease (7, 8), epilepsy (9, 10), multiple sclerosis (7, 11), anxiety disorders (12, 13), Crohn's disease (14, 15), glaucoma (16), nausea and vomiting (17) and analgesia (18). In 2018, the United States Food and Drug Administration (FDA) approved Epidiolex^®^, which is the first approved drug derived from Cannabis and is approved for the treatment of seizures in patients over 2 years of age suffering from two forms of drug-resistant epilepsy: Dravet and Lennox-Gastaut syndromes. In addition, the National Institute for Health and Care Excellence (NICE) in the UK approved the use of two cannabis-based medicines, Epidyolex^®^ and Sativex^®^, for the treatment of epilepsy and multiple sclerosis, respectively (19).

The potential therapeutic value of CBD in humans has made it an attractive option for use in animals (20). Moreover, CBD-supplemented pet food and treats are widely available for purchase by pet owners (21). However, the experimental evidence of the long-term safety of daily exposure in companion animals is currently limited. CBD is a small, lipophilic molecule, which could facilitate build-up in tissues with long-term feeding. The tissue-distribution of CBD and its endogenous receptors in dogs is not fully characterized, therefore there is a potential for adverse effects across different organs. A European Food Safety Authority (EFSA) panel recently assessed the available data pertaining to safety of CBD across a range of animal species. The panel found consistent evidence of potentially adverse effects on liver function and reproductive systems, though the specific measures varied by species and CBD formulation (22). These studies typically used levels of CBD many times higher than found in commercially available pet food and treats, however. Considering these factors, the EFSA panel concluded that a no observed adverse effect level (NOAEL, a dose at which there is substantial experimental evidence of no statistically and biologically significant increases in adverse effects) could not be established for CBD at this time (22).

Recent studies in dogs have investigated the short to mid-term safety of more moderate levels of CBD. Briefly, CBD concentrations of 2 mg/kg BW daily (23), 2 mg/kg BW twice daily (24, 25), 12 mg/kg BW daily (26), and up to 20 mg/kg daily (27), as well as escalating one-time dosing of CBD up to 62 mg/kg (28), were reportedly well-tolerated when orally administered to dogs. In these studies, some adverse effects were reported but classified as mild; these included gastrointestinal upset, hypersalivation and elevated serum alkaline phosphatase [ALP; (24–26, 28)].

The pharmacokinetic profile of CBD in dogs has been widely studied with the primary focus on 24-h pharmacokinetic analysis following a single dose (23–25). However, gaps in the knowledge surrounding longer term administration of CBD to dogs guided the two objectives of this study. First, to determine whether a 4 mg/kg BW daily oral dose of a broad-spectrum CBD (THC-free) distillate over a 6-month duration is tolerated, with a view to collect evidence in support of defining a NOAEL. Second, to evaluate the fasted plasma, fecal and urinary concentrations of CBD at regular intervals over the 6-month study period.

In general, monitoring markers of liver health are deemed vital for CBD studies due to the predominantly hepatic metabolism of cannabinoids (29). As such, alanine transaminase (ALT) was used as a primary measure in this study; ALT is documented to be the gold-standard marker of hepatocellular injury due to its high specificity and sensitivity in comparison to other liver enzymes (30, 31). ALP was also included as a secondary measure, as elevation can be indicative of liver injury and raised ALP levels have been previously reported after feeding of CBD to dogs over shorter periods (24, 26, 28, 32–34). In addition, a full suite of biochemical and hematological parameters, urinalysis, twice daily observational health and well-being checks, and fortnightly veterinary assessments were performed throughout the study.

## Materials and methods

### Animals and husbandry

This work was reviewed and approved by the Waltham Animal Welfare and Ethical Review Body and conducted under the authority of the Animals (Scientific Procedures) Act 1986. Forty healthy dogs took part in the study: 17 Labrador Retrievers (age = 1.4–9.4 y; weight = 19–36 kg), 8 Beagles (age = 1.2–6.6 y; weight = 11–18 kg), and 15 Norfolk Terriers (age = 1.4–4.4 y; weight = 4–8.5 kg). Study dogs were pair housed in kennels designed to provide free access to a temperature-controlled interior and an external pen at ambient temperature; dogs were provided with sleeping platforms at night. The dogs had access to environmentally enriched paddocks for group socialization and received lead walks and off-lead exercise opportunities during the day. Water was freely available throughout the trial. All dogs were trained and habituated to all procedures to minimize potential stress, and their behavior was monitored before, during and after each procedure.

Dogs were fed a standard commercial diet (Royal Canin^®^ Medium Adult Dry) twice daily. Food quantity offered was based on the individual's maintenance energy requirements (MER) and body condition score on a 9-point scale (36). Due to the duration of the study period and the number of dogs, two batches of the diet were used. Both batches of diet underwent nutritional analysis (Eurofins, UK). To ensure full compliance with the National Research Council (NRC 2006) essential nutrient requirements across the length of the study; 14 dogs received supplements with some meals (6 in the CBD group, 8 in the placebo group). The supplements added were Choline (Choline Chloride; Metabolics^®^), Selenium (Ionic selenium; Metabolics^®^) and Riboflavin (Riboflavin 5 Phosphate; Metabolics^®^). All dogs received a daily Greenies™ dental chew of appropriate size for their breed.

### CBD description and dosing

Hemp-derived distillate and placebo oils were acquired from Canopy Growth Corporation (Ontario, Canada) and processed by Kazmira LLC (Colorado, USA). The distillate was diluted with a food-grade sunflower oil and manufactured in soft gel capsules (bovine origin; RNA Corporation, Illinois, USA) to the following target concentrations (6, 10, 25, and 50 mg CBD), with each concentration indicated with a different colored capsule. The capsules were analyzed by a third-party laboratory for potency (Botanacor Laboratories, Colorado, USA) and to generate a certificate of analysis (CoA) for each batch concentration. The THC content of each batch was below the threshold of analytical detection (0.08%), as confirmed in the CoA. No other cannabinoids were detected except trace amounts (0.2 mg) of cannabidivarin in the 50 mg CBD capsules only. The placebo soft gel capsules were manufactured to match the conformity of the CBD-containing soft gel capsules, minus the CBD, to maintain the blinding of the study. The placebo capsules were also analyzed by the third-party laboratory to confirm they were void of detectable CBD. Each dog was provided with the combination of capsules to achieve as close to the target dose of 4 mg/kg BW per day as possible. Each capsule was placed in a Royal Canin^®^ Pill Assist moldable pocket and placed on the morning meal. Meals were monitored to ensure all dogs consumed the capsules.

### Study design

The study was conducted at Waltham Petcare Science Institute and was randomized, placebo-controlled, and blinded. Dogs were randomized and balanced across two parallel treatment groups: CBD and placebo. The parameters age, sex, breed, and housing location were considered when balancing the groups. The dogs were then separated into four staggers for logistical ease (10 dogs per stagger; 4–6 dogs in each treatment group), with a 1 week offset between stagger groups for trial initiation and collection of samples. To accurately dose CBD, dogs were weighed weekly. The targeted daily oral dose for each dog was 4 mg/kg BW with an acceptable range of 3.38–4.44 mg/kg BW.

The long-term study consisted of a baseline period of 8 weeks, during which all reference parameters were collected (biochemistry, hematology, urinalysis collected in the first week, twice daily unit observations, fortnightly vet checks). Subsequently dogs were administered a daily oral dose of either CBD or placebo as part of their morning food ration for 26 weeks (January to July) with measurements taken throughout (see Section Measures and analyses for details). Following the 26 weeks of daily administration, all dogs completed a 4-week washout period.

CBD is metabolized through the cytochrome-450 pathway and may have additive, synergistic, or antagonistic effects on other drugs that are metabolized through the same pathways (35). Due to a current lack of information on drug interactions between CBD and veterinary medications, a list of common veterinary drugs was assessed to help eliminate or minimize the risk of any drug interactions. These drugs were graded according to the theoretical or known risk of drug/CBD interaction based on their metabolism pathways as well as an assessment by the veterinary team on the severity of effect if an interaction should occur. No dog was denied appropriate veterinary treatment. When needed, low-risk drugs were provided following standard veterinary practice but were administered at least 6 h after CBD or placebo supplementation. For the medium and high-risk drugs, veterinarians formulated plans for treatment, which included alternative drug use as a first line of consideration, with the option for emergency unblinding if such a drug was deemed necessary.

### Measures and analyses

#### Blood-based measurements

At the end of baseline, and after 2, 4, 10, 18, and 26 weeks of intervention and at weeks 2 and 4 of washout, fasted (>12 h) jugular blood samples were collected (max 4.1 ml total volume). Lithium–heparin-treated blood was centrifuged and the resulting plasma used for the determination of standard biochemistry parameters; total protein, albumin, inorganic phosphate, alkaline phosphatase (ALP), alanine aminotransaminase (ALT), aspartate aminotransferase (AST), calcium, cholesterol, urea, creatinine, triglycerides, sodium, potassium, chloride and glucose, using an AU480 analyser (Beckman Coulter; USA). EDTA-treated blood was collected for the measurement of standard hematology parameters using a 3-part differential automated hematology analyser (Mythic 18 Vet, Orphée SA). Parameters measured were total leukocyte count, differentiated leukocyte counts as a number and percentage (lymphocytes, monocytes, and granulocytes), total erythrocyte count, hemoglobin concentration, haematocrit, mean corpuscular volume, mean corpuscular hemoglobin, mean corpuscular hemoglobin concentration, erythrocyte distribution width, platelet count, and mean platelet volume. EDTA-treated blood was also collected for CBD quantification. Serum clot activated blood was centrifuged and stored at 4 °C before sending to IDEXX Laboratories (UK) in a temperature-controlled box for analysis of total bilirubin, gamma-glutamyl transferase (GGT) and fasted bile acids using an AU5800 clinical chemistry analyser (Beckman Coulter; USA). Additionally, at baseline and 26 weeks, serum clot activated blood was used to evaluate markers of bone turnover: bone-specific alkaline phosphatase (BALP) and carboxy-terminal telopeptide cross-links (CTx) using MicroVue™ BALP ELISA kit (Quidel^®^, USA) and Serum CrossLaps CTX-I ELISA kit (Immunodiagnostic Systems Limited; UK), respectively. Both assays were performed according to the manufacturer's instructions on a Synergy HT plate reader (Agilent Technologies, USA) and have sensitivity limits of 0.7 U/L for BALP and 0.020 ng/mL CTX. Where described, reference ranges refer to those published by IDEXX Laboratories (UK).

#### Urinalysis

At the end of baseline, after 4, 10, 18, and 26 weeks of intervention and at 4-week washout, urine was collected (min 3 ml total volume) using a free-catch method with a uripet (Fisher Scientific; UK). Urine specific gravity was measured using a refractometer (J.A.K. Marketing Ltd, UK) and glucose, bilirubin, ketone, specific gravity, blood, pH, protein, urobilinogen, nitrite, and leukocytes were analyzed using the Status Plus Analyser with Multistix^®^ 10SG urine test strips (Siemens Healthcare Limited; UK). An aliquot of urine was also processed for CBD quantification.

#### Feces collection

At the end of baseline, after 4, 10, 18, and 26 weeks of intervention, and at 4-week washout a single fresh fecal sample was collected, and CBD levels were quantified.

### CBD extraction and mass spectrometry analysis

Extraction and analyses of plasma were performed as previously described (28). Briefly, an Agilent 1,290 liquid chromatograph (LC) coupled with a 6,460 Triple Quadrupole (QQQ) mass spectrometer (MS), operated by Masshunter software (Agilent; USA) was used for analysis. The LC column used was Kinetex 2.6 μm Phenyl-Hexyl 100 A, 50 x 2.1 mm, along with a guard column, X3 SecurityGuard ULTRA Cartridges UHPLC Phenyl (Phenomenex, Cheshire, UK). The mobile phase was delivered at a flow rate 0.4 mL/min and the gradient parameters were as follows (solvent A was 0.1% formic acid in UHQ water, solvent B was 0.1% formic acid in acetonitrile): 0 min: 30% B, 5.3 min: 95% B, 6.3 min: 70% B. The scanning conditions were in multiple reaction monitoring. Cannabidiol (CBD) and Cannabidiol-D3 (CBD-d3, used as an internal standard) certified reference materials were obtained from Sigma-Aldrich (Dorset, UK). Methanol, acetonitrile and acetonitrile with 0.1% formic acid (v/v) were obtained from Fisher (Loughborough, UK). For urine samples, the internal standard (300 μL of 40 ng/mL, CBD-d3) was aliquoted into 100 μL of canine urine and vortexed for 5 s. Samples were centrifuged (12,000 rpm for 5 mins) and 325 μL of the supernatant was aliquoted into a labeled glass amber vial containing 650 μL 0.1% formic acid in water. A screw cap was placed on each vial, which was vortexed again (5 s) and analyzed as described. For feces samples, the internal standard (750 μL of 300 ng/mL, CBD-d3) was aliquoted onto samples (0.25 ± 0.01 g, canine feces) and vortexed for 30 min. Samples were centrifuged (5,000 rpm for 10 min) and 390 μL of the supernatant was aliquoted into a labeled Eppendorf tube containing 780 μL of 0.1% formic acid in water, vortexed (5 s) and centrifuged for a second time (14,000 rpm for 10 min). The supernatant was aliquoted to a labeled glass amber vial, a screw cap was placed on each vial and analyzed as described.

### Health evaluations

For the duration of the study period, daily food intake and weekly body weight and body condition scoring (using a 9-point scale) (36) were recorded and monitored. General health observations of the dogs were performed twice daily (AM and PM) by blinded pet caretakers to record and monitor any observable adverse effects, for example gastrointestinal (GI) upset, hypersalivation or ataxia. A full veterinary health assessment was performed fortnightly by a blinded veterinarian. A general dog Quality of Life (QoL) survey was completed at the end of every day for every dog (37). This was completed by a blinded experienced handler who was familiar with the daily behavior of the dogs, with each dog scored across 5 domains (happy, energetic, mobile, relaxed, and sociable). Blood data (biochemistry and hematology) for each dog was reviewed by a blinded veterinarian after each sampling point to assess general health.

### Statistical analysis

All statistical analysis was carried out using the open-source statistical software R, version 4.1.2 (38). The sample size for this study was determined through a power analysis by simulation. Adult dog ALT measurements from Waltham Petcare Science Institutes' internal database were used to estimate the between breed, between animal and within animal variance components. Using these variance components, data sets were simulated in the study design for a range of dog numbers. For each “number of dogs,” 1,000 data sets were created, and the primary analysis as described was applied to each. The power was reported as the percentage of the 1,000 data sets where equivalence could be declared at 2-fold limits, given that no difference between the diet groups or time points had been induced. The sample size recommended was the number of dogs estimated to achieve 80% power, inflated by ~25% for potential study dropout. The minimum sample size determined was 32 dogs, with 40 dogs selected to begin the study.

### Primary objective (ALT)

For ALT, a linear mixed model was fitted to the log_10_ activity level, with treatment group, time point and their interaction as categorical fixed effects, and animal nested in breed as the random structure. The residuals of the model were visually assessed for normality and homogenous variance. Within each treatment group, comparisons between baseline and each subsequent time point were tested, and at each time point a comparison between treatment groups was also tested. All comparisons were tested for equivalence at 2-fold limits using TOST (two one sided tests) at a 5% significance level, adjusted for family-wise error-rate. Fold changes with 95% confidence intervals are reported alongside their *p*-values. Back-transformed estimates of the mean (raising 10 to the power of the raw estimates) and 95% confidence intervals are also provided for each treatment/timepoint.

### Secondary parameters (biochemistry, hematology, urinalysis)

For secondary parameters, a linear mixed model was fitted with the same fixed and random effect structure as for ALT with the same planned comparisons performed. Assumptions of normality were assessed through visual inspection of residuals. If this assumption was deemed to be violated, the response variable was log_10_ transformed; where this was not possible, the response variable was ranked transformed as a robust alternative. Relevant estimates of the differences with 95% confidence intervals are reported alongside their *p*-values, using a family-wise adjusted 5% significance level. Estimates of the means and 95% confidence intervals, or medians and interquartile ranges, are also provided for each treatment/timepoint. Pearson's correlation analysis was performed between ALP and BALP at 26 weeks, to assess whether these parameters are potentially linked; the associated *p*-value is reported to assess whether the correlation is statistically significant.

### Quality of life

For each of the five Quality of Life domains, all scores were calculated on a 1–7 scale as previously described (37). Score were fit to a linear mixed effects model with diet as the fixed effect and individual dog as the random effect. The means and 95% confidence intervals were reported. The difference in means, confidence intervals and *p*-values were calculated. Due to comparisons being made in 5 domains, a Bonferroni adjustment to the alpha level was used, resulting in a test level of *p* ≤ 0.01.

### CBD concentration

To assess CBD concentration in the plasma, feces and urine, line-plots are produced for each dog and arranged by treatment group. Additionally, for plasma the observations are plotted by group/timepoint, with means and 95% confidence intervals plotted for the CBD cohort at timepoints 2, 4, 10, 18, and 26 weeks. Analysis of these figures is reported descriptively. Pearson's correlation analysis was performed between CBD plasma concentration and ALP levels to assess whether these parameters are linked. The associated *p*-value is reported to assess whether the correlation is statistically significant.

## Results

**Figure 1:**
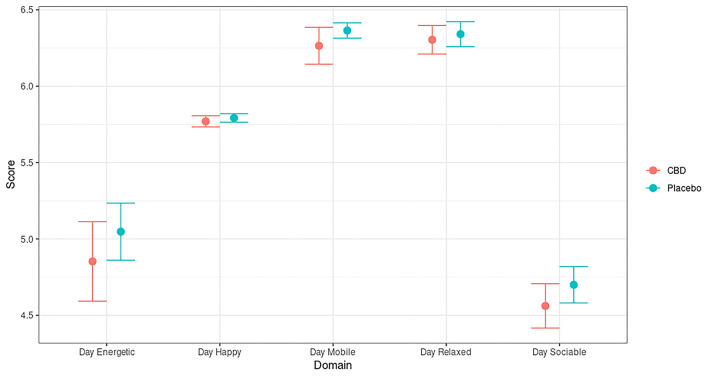
Daily quality of life (QoL) assessment across five domains in dogs dosed with CBD (red) and placebo (blue), where a score of 7 is a maximum and 1 is a minimum possible value for each domain. Values represent mean and 95% confidence intervals of 1,001 surveys from the CBD group, and 1,097 surveys from the placebo group.

All 40 dogs completed the study with no CBD or placebo capsule refusals. Dogs were fed to maintain a healthy body weight and BCS and no deviations were noted (data not shown). CBD was fed to a target of 4 mg/kg BW/day with the dosing range throughout the study being 3.38–4.44 mg/kg/BW and the average concentration being 3.99 mg/kg/BW/day. No adverse effects were detected during the twice daily observational health and well-being checks or fortnightly veterinary examinations. A daily QoL survey completed by the dog's care providers (37) found no significant differences between the CBD and placebo groups in any of the five domains assessed (all *p* > 0.05, Figure 1), indicating that the general well-being of healthy dogs was not negatively impacted by CBD administration.

### Hematology and biochemistry

All mean hematology values across both the CBD and placebo groups were within reference ranges at all times measured (see Supplementary Data S1). For some hematology measures, significant differences were observed during CBD/placebo administration when compared to baseline within the treatment group (effect of time, *p* < 0.05). However, there were no significant differences detected between the CBD and placebo groups (*p* > 0.05).

**Figure 2:**
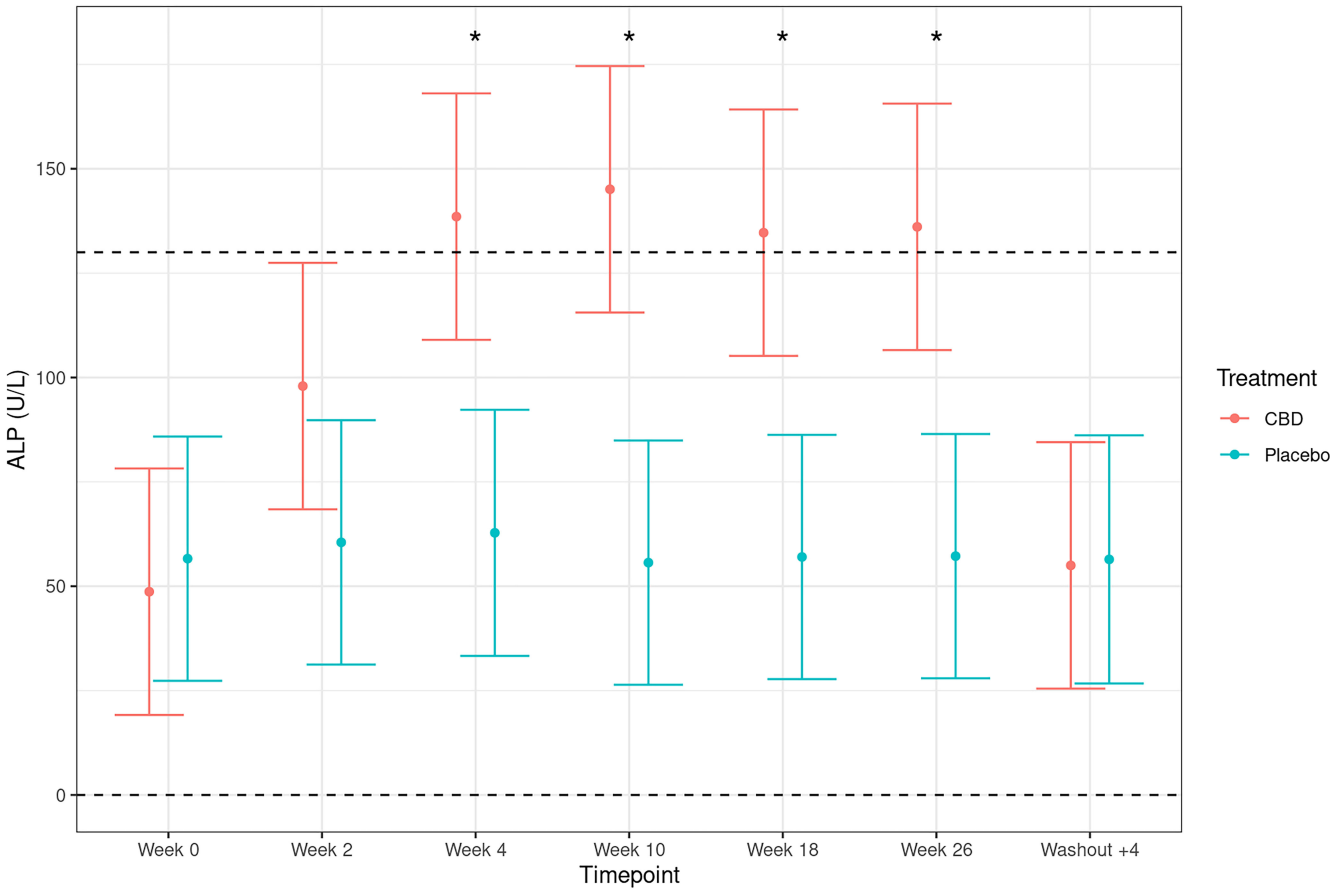
Plasma activity levels of total alkaline phosphatase (ALP; U/L; mean and 95% confidence intervals) in dogs dosed with CBD (red) and placebo (blue). Week 0 depicts the baseline measure before daily oral dosing of CBD/placebo. Dotted line depicts upper and lower reference ranges as specified by IDEXX Laboratories. *Depicts significant differences between the experimental groups (*p* < 0.001).

Mean values for serum biochemistry parameters alkaline phosphatase (ALP), protein and calcium were outside of reference ranges at different timepoints during the study. Although unlikely to be clinically relevant due to a lack of concomitant clinical signs, mean calcium levels were below the reference range by 0.04 mmol/L at one timepoint (week 18) in CBD-treated dogs (see Supplementary Data S2a). Mean protein levels in CBD treated dogs fell below the reference range by 0.8, 1.15, 1.23, 2.19, and 1.1 g/L at 2, 4, 10, 18, and 26 weeks, respectively (see Supplementary Data S2b). ALP activity levels were above the reference range at 4, 10, 18, and 26 weeks by 8.5, 15.1, 4.7, and 6.1 U/L, respectively, in CBD-treated dogs (Figure 2). Furthermore, mean ALP activity levels were significantly higher in CBD-treated dogs in comparison to placebo-treated dogs at 4, 10, 18, and 26 weeks (treatment by time interaction, *p* < 0.001), with mean activities returning to that of placebo-treated dogs 4 weeks after ceasing CBD administration (washout +4). Eleven of the 20 dogs receiving CBD (but 0/20 receiving placebo) showed ALP activity levels that were elevated above the clinical reference range (see Supplementary Data S3). One Labrador in particular (Labrador 17) showed a 5-fold increase from baseline at week 10. Importantly, this was not accompanied by any other elevated clinical parameters and dropped to a 3-fold increase at the next sample point (week 18). All dogs that showed elevated ALP during CBD administration returned to baseline values after the 4-week washout.

**Figure 3:**
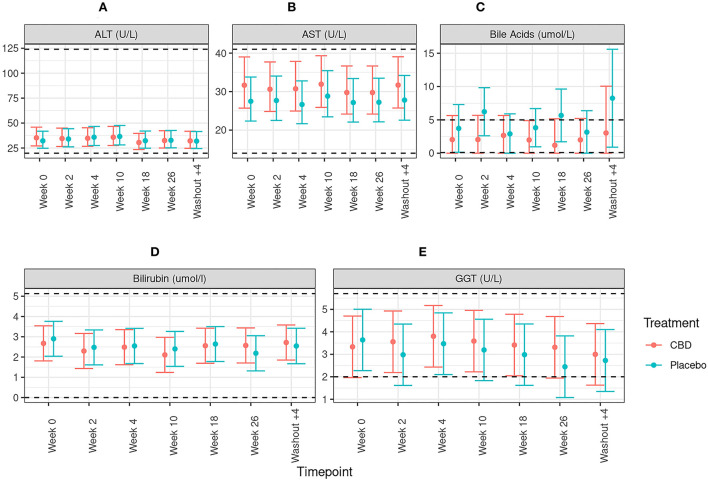
Means and 95% confidence intervals of **(A)** alanine transaminase activity (ALT; U/L), **(B)** aspartate aminotransferase activity (AST; U/L), **(C)** Bile acid concentration (umol/L), **(D)** bilirubin concentration (umol/L) and **(E)** gamma-glutamyl transferase activity (GGT; U/L) in dogs dosed with CBD (red) and placebo- (blue). Week 0 depicts the baseline measure before daily oral dosing of CBD/placebo. Dotted line depicts upper and lower reference ranges as specified by IDEXX Laboratories.

**Figure 4:**
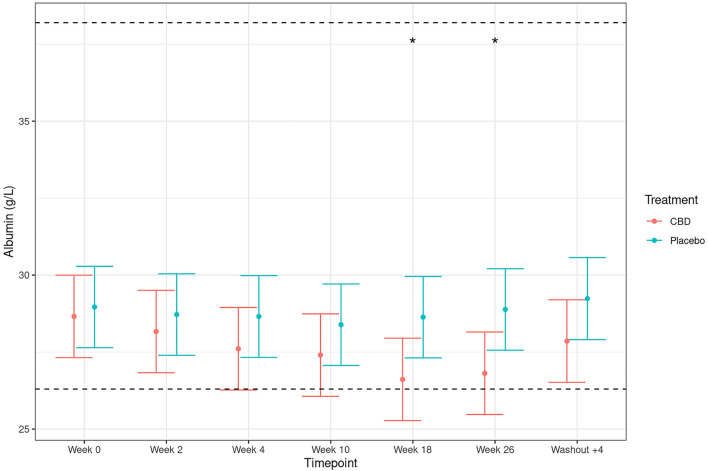
Plasma concentrations of albumin (g/L; mean and 95% confidence intervals) in dogs dosed with CBD (red) and placebo (blue). Week 0 depicts the baseline measure before daily oral dosing of CBD/placebo. Dotted line depicts upper and lower reference ranges as specified by IDEXX Laboratories. *Depicts significant differences between the experimental groups (*p* < 0.005).

All other measures of liver function; alanine aminotransferase (ALT), aspartate aminotransferase (AST), gamma-glutamyl transferase (GGT), total bilirubin and fasted bile acids, showed no significant difference between CBD- and placebo-treated dogs at any time point (all *p* >0.05, Figure 3). Although within physiological reference range, there was a significant difference between treatment groups for albumin at weeks 18 and 26, with mean concentrations around 2 g/L lower in CBD-treated dogs (Figure 4; treatment by time interaction *p* < 0.001). For the remaining plasma biochemical parameters, there were no significant differences between treatment groups (see Supplementary Data S4).

### BALP and CTX

**Figure 5:**
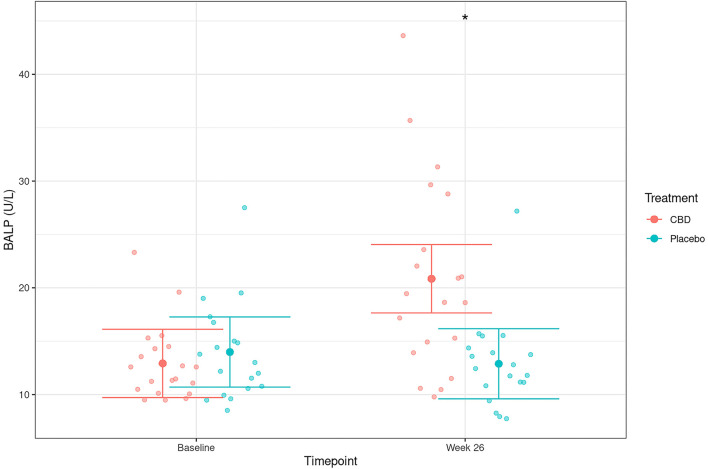
Serum activity levels of bone-specific alkaline phosphatase (BALP; mean and 95% confidence intervals) in dogs dosed with CBD (red) and placebo (blue) at baseline and 26 weeks. *Depicts significant differences between the experimental groups (*p* < 0.001).

**Figure 6:**
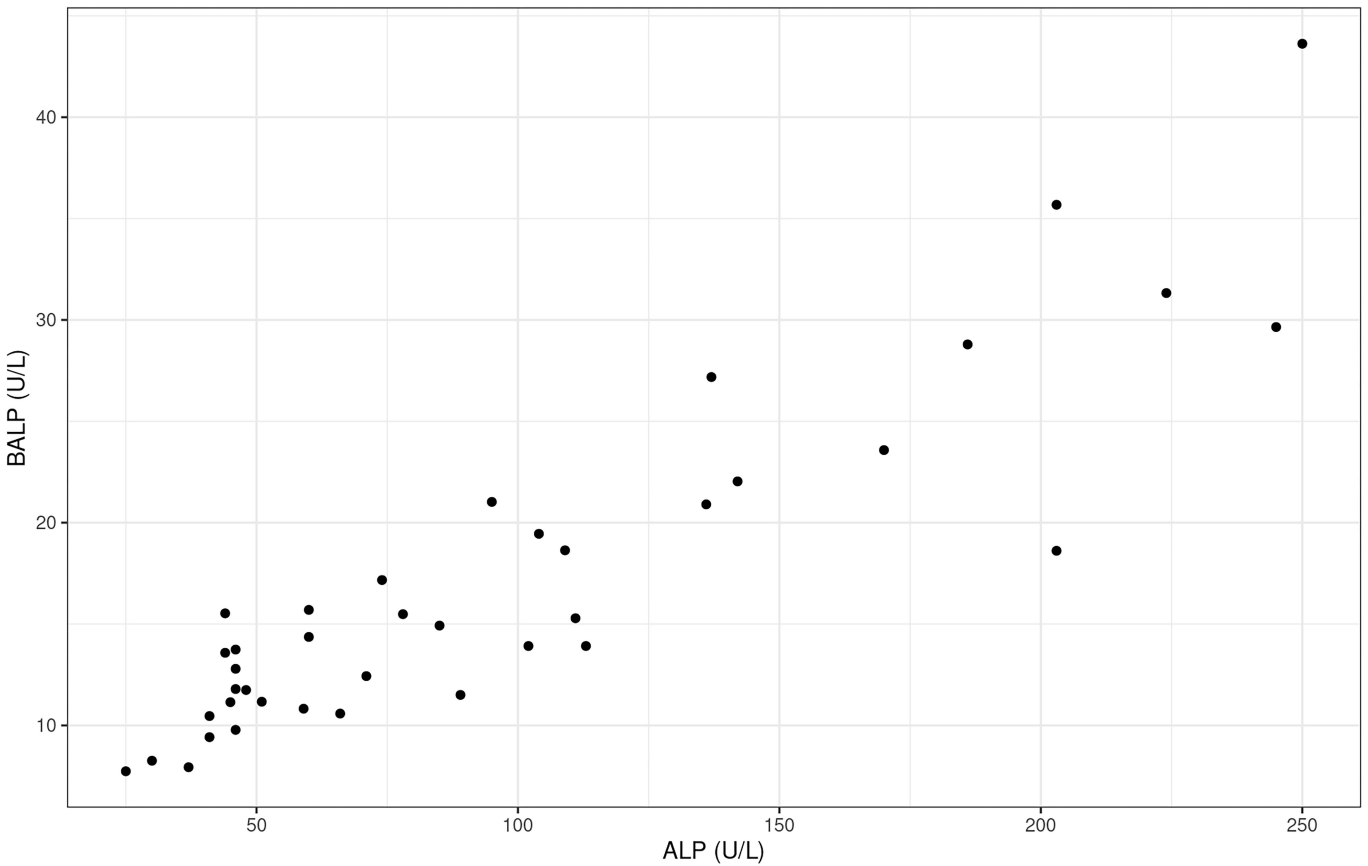
Scatter plot to depict the strong positive correlation between total activity levels of bone-specific alkaline phosphatase (BALP; U/L) and alkaline phosphatase (ALP; U/L) in dogs dosed with CBD at the 26-week time point (*r* = 0.9).

To gain a better understanding of the tissue source of ALP, BALP activity, the bone specific isoform of ALP, and CTX were measured at baseline and at the end of the 26-week administration period. No differences were observed in CTX, either within or between groups (all *p* > 0.05, data not shown). However, significantly increased BALP activities were observed after 26 weeks in CBD dosed dogs in comparison to placebo dogs (Figure 5; treatment by time interaction *p* < 0.001). Furthermore, there was a strong, significant positive correlation between ALP and BALP activity at 26 weeks in CBD dosed dogs (*r* > 0.9, *p* < 0.001; Figure 6).

### Plasma, urine and fecal CBD concentrations

**Figure 7:**
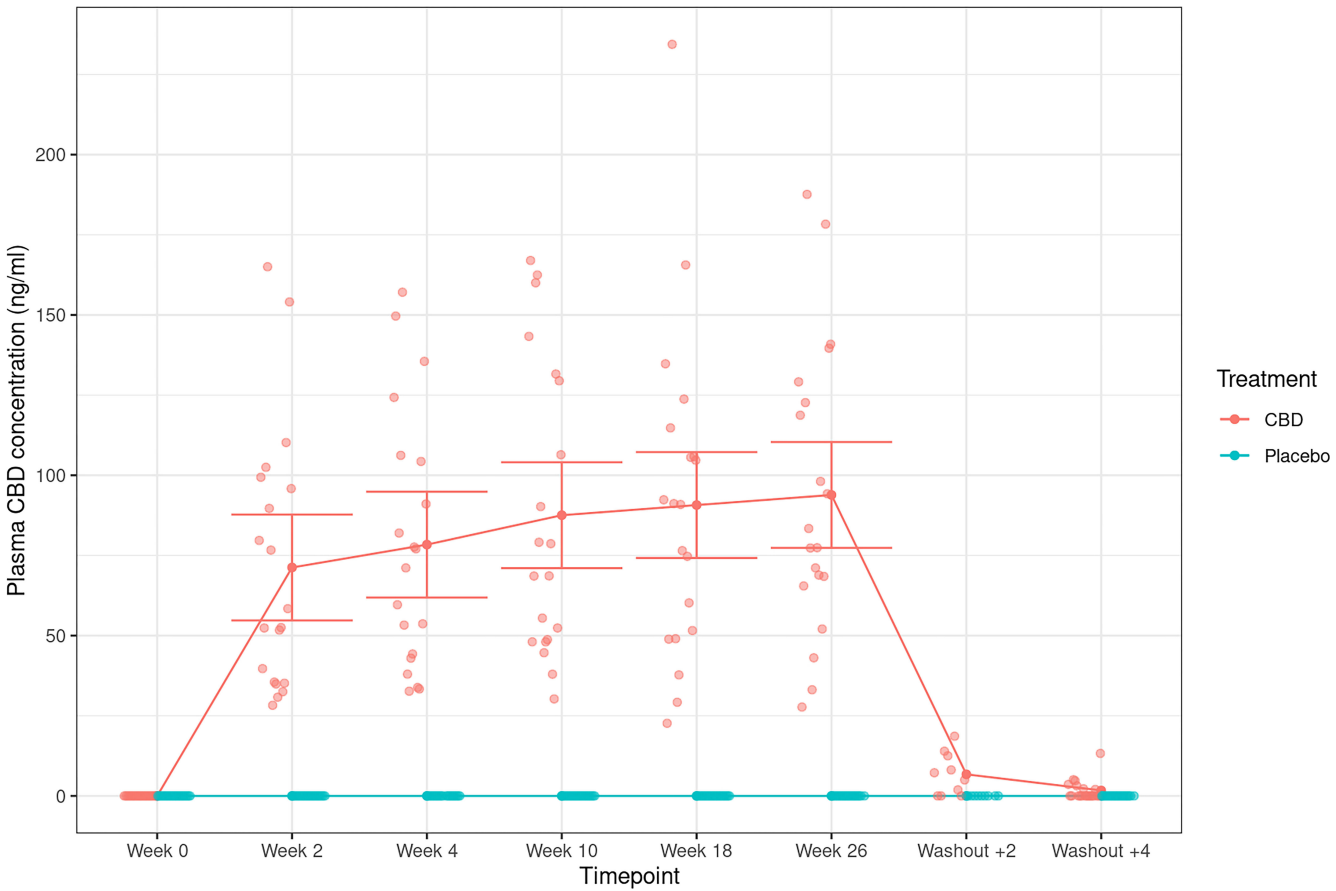
Plasma CBD concentrations (ng/ml; mean and 95% confidence intervals) in dogs dosed with CBD (red) and placebo (blue) at each study time point. Week 0 depicts the baseline. Solid red line shows the mean concentration across the time points in CBD-dosed dogs.

**Figure 8:**
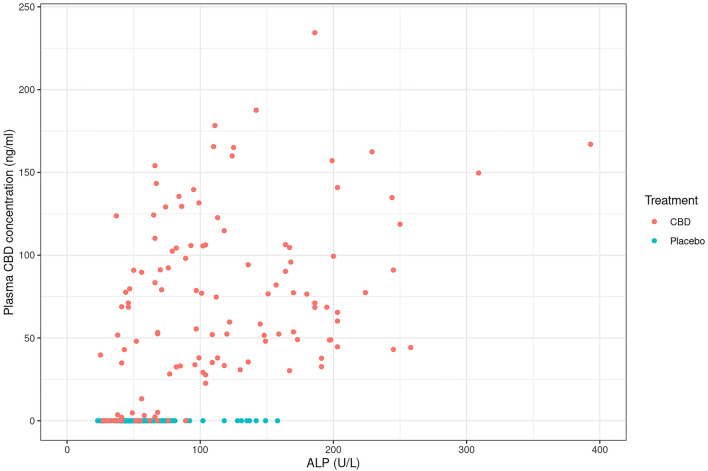
Scatter plot to depict a weak positive correlation between plasma CBD concentration (ng/ml) and alkaline phosphatase (ALP; U/L) in CBD-dosed dogs when all time points are combined (*r* = 0.469).

Mean fasted plasma CBD levels showed a steady increase over time during CBD exposure, whilst a sharp decline following the 2- and 4-weeks washout was observed (Figure 7). Comparing across CBD fed dogs there was a wide variation in CBD concentration range at each time point (2-weeks: 28–165 ng/ml; 4-weeks: 33–157 ng/ml; 10 weeks: 31–167 ng/ml; 18-weeks: 23–234 ng/ml; 26-weeks: 28–188 ng/ml). Individually, Norfolk terriers tended to have lower plasma CBD concentrations than Labradors (see Supplementary Data S5); however the study was not powered to statistically confirm a breed difference. As ALP was elevated in dogs that received CBD, the data was plotted to determine if there was any correlation between the two parameters. There was a weak albeit significant, positive correlation between CBD plasma concentration and ALP levels (Figure 8; *r* = 0.469, *p* < 0.001). Similar to the plasma concentrations, CBD excretion in the feces was widely variable between the dogs. Concentrations in the urine were notably lower and only detectable in 12 of the 20 dogs (see Supplementary Data S6).

### Urinalysis

For urinalysis, no significant differences were identified between treatment groups for urine-specific gravity and pH (all *p* > 0.05, Table 1). Additional urinary parameters measured by the test strips (glucose, bilirubin, ketones, blood, protein, uroliths, nitrogen, leukocyte), were reviewed by a qualified, blinded, veterinarian after each time point and no clinical concerns were highlighted (data not shown).

**Table 1:** Mean and 95% confidence intervals of urine pH measured by dipstick, and urine specific gravity measured by refractometer, in dogs dosed with CBD and placebo.

**Time**	**Treatment**	**Urine**	**Urine specific**
**point**	**group**	**pH**	**gravity**
		**(mean ±CI)**	**(mean ±CI)**
Week 0	CBD	6.357 ± 1.11	1.025 ± 0.01
	Placebo	5.97 ± 1.10	1.032 ± 0.01
Week 4	CBD	6.717 ± 1.11	1.033 ± 0.01
	Placebo	6.392 ± 1.11	1.034 ± 0.01
Week 10	CBD	6.642 ± 1.11	1.032 ± 0.01
	Placebo	6.911 ± 1.11	1.035 ± 0.01
Week 18	CBD	6.555 ± 1.11	1.033 ± 0.01
	Placebo	6.72 ± 1.10	1.03 ± 0.01
Week 26	CBD	6.545 ± 1.10	1.033 ± 0.01
	Placebo	6.658 ± 1.10	1.033 ± 0.01
Washout +4	CBD	6.158 ± 1.10	1.031 ± 0.01
	Placebo	6.108 ± 1.10	1.034 ± 0.01

Week 0 depicts the baseline measure before daily oral dosing of CBD/placebo.

## Discussion

The aim of this current study was two-fold. First to demonstrate tolerance of a once-daily oral dose of a broad-spectrum CBD (THC-free) distillate provided at 4 mg/kg BW over a 6-month period to healthy adult dogs. Second to quantify the level of CBD in the fasted plasma, urine, and feces over the same period. The study demonstrated that a daily oral dose of CBD at this concentration and duration was well-tolerated by clinically healthy adult dogs.

All hematology and urinalysis measures remained within the reference range for both placebo and CBD groups at all sample points with no significant differences seen between the groups. Biochemistry values were also generally unremarkable with only transient changes seen in calcium and protein measures with no associated clinical signs. We do not deem these to be of concern, especially when evaluated in a broader clinical context (30). However, changes were observed in ALP levels where the CBD group had a significant elevation, with the levels returning to baseline after the 4-week washout period. This finding is not unexpected, as elevated ALP activity has been previously observed in CBD studies with dogs (24, 26, 28, 32–34). In past publications, increases in ALP observed with CBD exposure was considered to be due to induction of cytochrome p450-mediated oxidative metabolism in the liver, as suggested after long-term exposure to cannabis (39, 40). Increased ALP activity is a reasonably sensitive indicator of hepatobiliary changes in dogs but still has the lowest organ specificity of the routinely used liver enzymes (41). Although the transient elevation of ALP could be suggestive of impaired liver function, it is clinically uninformative when interpreted in isolation of other liver parameters. As such, a comprehensive panel of liver markers in the blood were analyzed simultaneously throughout the study (ALT, AST, ALP, GGT, bilirubin, fasted bile acid, albumin, and cholesterol). No significant differences were observed between the CBD- and placebo-fed dogs at any time point for ALT, AST, GTT, bilirubin, fasted bile acid, or cholesterol. Significant differences in albumin levels were observed between the two groups at 18 and 26 weeks, however the mean values were within the reference range, so this observation is unlikely to be clinically relevant. Taken together, alongside the return to baseline after cessation of CBD feeding, the ALP increase observed was not assumed to be a clinically relevant biomarker for impaired liver health in these dogs.

Although ALP is commonly associated with liver health, other ALP isoenzymes are expressed in bone, kidney, placenta and intestines. The serum half-life of placental, kidney and intestinal ALP is <6 min (42) and therefore sustained total ALP activity levels in the blood likely originates from either the liver, bone or as a result of corticosteroid induction (30). This study identified a significant elevation in bone-specific ALP after 26 weeks of daily CBD exposure. The same dogs that had the largest increase in ALP also had the largest increase in BALP. The strong positive correlation between elevations in total ALP and BALP suggests that the rise in total ALP could be, at least partly, a consequence of increased osteoblastic activity, though further research will be required to confirm this directly. Non-specific cross reactivity with other isoenzymes of ALP has been reported when using immunoassays for evaluation of BALP (43, 44). However, the methodology used in the current study has been validated for use in canines (45) and tested for specificity to canine BALP (46, 47). In contrast, a marker of osteoclastic activity (CTX) did not display a significant increase in the CBD group over the same period. Taken together these data may indicate a possible role for CBD in supporting bone health in dogs, as has been reported in other species. In rats, CBD was shown to improve fracture healing (48) and bone mineral density (49). Furthermore, CBD induced differentiation in two kinds of human osteoblast-like cell lines, demonstrating its potential to promote bone formation (50), as well as promoting osteogenic differentiation in bone marrow mesenchymal stem cells (51).

A full pharmacokinetic assessment of CBD in dogs was not evaluated in this study as this has been characterized elsewhere (23–26). However, there is currently limited data illustrating changes in the concentration of CBD in the plasma with repeated long-term daily administration. This study shows that mean CBD levels steadily increased in plasma during the 26-week feeding period, which could be indicative of a mild accumulation, before decreasing to negligible levels 4 weeks after the final dose. The range of CBD levels in plasma during the feeding period (~25–275 ng/ml) was comparable to the maximum levels recorded in a pharmacokinetic analysis after feeding four Beagles 4 mg/kg per day CBD for 4-weeks (106–249 ng/ml) (26). It was, however, lower than has been observed across a 12-week study of dogs dosed at 5 mg/kg per day (~130–940 ng/ml). This may reflect differences in CBD bioavailability or in the blood sampling schedule, though the latter study involved dogs with idiopathic epilepsy that were also provided anti-convulsant drugs (52).

Given the large variation in the plasma concentration between individual dogs, further work using a greater number of dogs will be required to establish the dynamics of CBD over a long period of feeding. Variability across dogs has been documented in previous studies (27, 28) and may be indicative of individual and/or breed-specific differences in cannabinoid metabolism or absorption rates. Indeed, in this study the larger dogs (the Labrador retrievers) tended to have higher CBD plasma concentrations than the smaller Norfolk terriers, with the largest difference being over 200 ng/ml after 18 weeks. Consequently, future studies into the efficacy of CBD in dogs may benefit from individualized dosing to achieve more comparable plasma concentrations. One study demonstrated this in osteoarthritic dogs by reviewing CBD levels every 2 weeks throughout a 90-day study and adjusting the dose as needed to achieve a beneficial effect (53). The dogs requiring the highest doses were Cavalier King Charles spaniels, supporting the hypothesis that dog breeds may metabolize CBD in a different manner. Combined genetic and metabolomic studies will be required to test this hypothesis further, but other instances of dog breed-specific differences in the cytochrome-450 pathway have been reported. For example, non-coding genetic variants that reduce expression of the *CYP2B11* gene is the likely mechanism responsible for the slow clearance that Greyhounds display for some anesthetics (54).

This is the first long-term study in dogs to investigate the elimination routes of CBD in urine and feces. Similar to plasma, concentrations varied between the dogs. In general, significantly greater amounts of CBD were eliminated with the feces than the urine; this is unsurprising due to incomplete absorption of the cannabinoid after oral administration. This is also broadly consistent with data from humans, where 33 and 16% of the total dose of CBD was calculated to be excreted in the feces and urine, respectively (55). Short-term studies conducted in dogs have shown that not all dogs receiving CBD have detectable levels of CBD excreted in their urine (56, 57). This was also observed in the current study, where only 60% of dogs had detectable levels of CBD in urine, after 26 weeks of daily feeding. The variation observed in both urine and fecal samples may be partly due to the longer time window in which they were collected (due to a reliance on natural voiding). In addition, metabolites of CBD, which are expected to be found in urine in higher concentrations than CBD itself, were not investigated. Consequently, a complete picture of how dogs eliminate CBD during long-term feeding remains to be determined.

In addition to the broadly comparable clinical pathology and biochemical measures already discussed, each dog was evaluated with a fortnightly physical examination performed by a blinded veterinarian, twice daily welfare checks by blinded caretakers, and daily quality of life surveys. Collectively, these assessments revealed no differences between the health and well-being of CBD and placebo dosed dogs.

In conclusion, the CBD (THC-free) distillate used in this study is well-tolerated by dogs, which should contribute data toward the future establishment of a NOAEL (22) Notwithstanding this, caution should be exercised in generalizing the conclusion from this long-term study to CBD-containing products more broadly. Many factors affect the constitution of cannabinoids and terpenes in cannabis plant distillates, ranging from cultivar to temperature, and soil type to extraction process (58–61). Furthermore, this study was conducted on clinically healthy adult dogs, in a controlled environment, and was designed to minimize the risk of any drug interactions. Additional studies will be required to understand any risks associated with the interaction of CBD and commonly prescribed veterinary medications, as well as to understand how disease processes would affect the tolerance of CBD.

## Data availability statement

The raw data supporting the conclusions of this article will be made available by the authors, without undue reservation.

## Ethics statement

The animal study was reviewed and approved by the Waltham Petcare Science Institute Animal Welfare and Ethical Review Body.

## Author contributions

SB, AH, DL, PW, AB, SY, and LH: conceptualization and design. SB and KP: methodology. DW: statistical analysis, data curation, and visualization. SB: investigation and writing—original draft preparation. SB, SY, AB, DW, KP, LH, DL, and PW: writing—review and editing. LH, DL, and PW: supervision. All authors have read and agreed to the published version of the manuscript.

## Funding

The study was funded by Mars Petcare.

## Conflict of interest

SB, SY, AB, LH, DW, AH, KP, PW, and DL are employees of Mars Petcare, a manufacturer of pet food and provider of veterinary services.

## Publisher's note

All claims expressed in this article are solely those of the authors and do not necessarily represent those of their affiliated organizations, or those of the publisher, the editors and the reviewers. Any product that may be evaluated in this article, or claim that may be made by its manufacturer, is not guaranteed or endorsed by the publisher.

## Supplementary material

Supplementary Table (S1) Full suite of hematology parameters measured at each time point for both CBD- and placebo- fed dogs. Bold reflects significance within the treatment group at that time point and b indicates significance between the treatment groups at that time-point.

Supplementary Figure 1 (S2) Plasma concentrations (mean and 95% confidence intervals) of calcium [**(A)**; mmol/L] and protein [**(B)**; g/L] in dogs dosed with CBD (red) and placebo (blue). Week 0 depicts the baseline measure before daily oral dosing of CBD/placebo. Dotted line depicts upper and lower reference ranges as specified by IDEXX Laboratories.

Supplementary Figure 2 (S3) Plasma activity levels of total alkaline phosphatase (ALP; U/L) at each study time-point, in each of 20 individual dogs receiving CBD. Breed of each dog is detailed above each individual graph. Dotted line depicts upper and lower reference ranges as specified by IDEXX Laboratories.

Supplementary Table 2 (S4) Full suite of biochemical parameters measured in the plasma at each time point for both CBD- and placebo- fed dogs. Bold reflects significance within the treatment group at that time point and b indicates significance between the treatment groups at that time-point.

Supplementary Figure 3 (S5) Plasma levels of CBD (ng/ml) at each study time-point, in each individual dog receiving CBD. Breed of each dog is detailed in the legend above each graph. Week 0 depicts the baseline.

Supplementary Figure 4 (S6) CBD concentrations in feces [**(A)**; ng/g] and urine [**(B)**; ng/ml] for each CBD-dosed dog at each study time-point. Week 0 depicts the baseline.
