# Supplementary material for: Long-term daily feeding of cannabidiol is well-tolerated by healthy dogs
Source: Front Vet Sci. 2022 Sep 21;9:977457. doi: 10.3389/fvets.2022.977457 (PMC9533147; doi:10.3389/fvets.2022.977457)
Supplement: Supplementary Table (S1) — Full suite of hematology parameters measured at each time point for both CBD- and placebo- fed dogs. Bold reflects significance within the treatment group at that time point and b indicates significance between the treatment groups at that time-point. [file Data_Sheet_1.PDF]

	Parameters	Week0:CBD	Week0:Placebo	Week2:CBD	Week2:Placebo	Week4:CBD	Week4:Placebo	Week10:CBD	Week10:Placebo	Week18:CBD	Week18:Placebo	Week26:CBD	Week26:Placebo	Wash4:CBD	Wash4:Placebo
1	RBC	7.07±0.15	6.97±0.14	7.05±0.05	7.01±0.07	7.03±0.05	6.92±0.07	6.89±0.05	6.95±0.07^b	6.69±0.06^b	7.26±0.08	6.8±0.05^b	6.92±0.07^b	6.77±0.05	7.01±0.08
2	HGB	16.24±0.36	16.2±0.3	16.23±0.13	16.06±0.18	16.15±0.12	15.88±0.18	15.8±0.12	15.91±0.18^b	15.36±0.14^b	16.7±0.19	15.94±0.13	15.83±0.18^b	15.91±0.13	16.04±0.18
3	HCT	45.04±1.22	45.22±0.89	45.12±0.36	44.51±0.51	45.12±0.35	43.89±0.5	44.5±0.35	44.07±0.51	42.97±0.4^b	46.26±0.56	43.58±0.36	43.84±0.51^b	43.6±0.36	44.43±0.51
4	MCV	**63.55±0.73**	**64.93±0.44**	63.89±0.1	63.34±0.15	64.08±0.1^b	63.25±0.15	64.5±0.1^b	63.34±0.15^b	**64.18±0.12**^{b}	**63.55±0.16**^{b}	63.99±0.1^b	63.2±0.15	64.33±0.1	63.3±0.15
5	MCH	22.97±0.17	23.27±0.15	23.02±0.04	22.91±0.06	22.97±0.04	22.94±0.06	22.95±0.04	22.89±0.06	22.97±0.05	22.98±0.07	23.45±0.04^b	22.87±0.06^b	23.52±0.04	22.9±0.06
6	MCHC	36.11±0.22	35.83±0.16	36.02±0.07	36.14±0.11	35.83±0.07	36.23±0.1	35.56±0.07^b	36.14±0.11^b	35.78±0.08^b	36.14±0.11^b	36.62±0.07^b	36.15±0.11^b	36.54±0.07	36.16±0.11
7	RDW-SD	34.63±0.3	35.3±0.39	34.55±0.12	34.83±0.17	34.62±0.12	34.59±0.17	35.05±0.12	34.37±0.17	**34.36±0.14**	**35.19±0.19**	33.97±0.12^b	34.29±0.17^b	**33.62±0.12**	**35.17±0.18**
8	RDW-CV	16.68±0.21	16.27±0.3	16.42±0.13	17.01±0.18	16.37±0.12	16.73±0.18	16.33±0.12	16.6±0.18	15.63±0.14^b	17.47±0.2	16.77±0.13	16.35±0.18	16.33±0.13	17.3±0.18
9	RETCount	37.82±5.54	40.56±5.68	35.47±2.59	37.8±3.71	30.94±2.55	41.55±3.65	35.45±2.56	36.08±3.72	31.24±2.89	48.17±4.03	42.45±2.59	25.79±3.72	35.83±2.59	38.48±3.74
10	RETPercentage	0.54±0.08	0.59±0.09	0.5±0.04	0.55±0.05	0.44±0.04	0.61±0.05	0.51±0.04	0.53±0.05	0.47±0.04	0.68±0.06	0.63±0.04	0.37±0.05	0.53±0.04	0.55±0.05
11	IRF	20.82±2.53	24.94±1.9	22.92±1.26	20.22±1.8	22.99±1.24	20.14±1.78	23.12±1.25	16.12±1.81	21.98±1.4	19.51±1.96	26.49±1.26^b	20.34±1.81^b	21.6±1.26	19.26±1.82
12	LFR	79.18±2.53	75.06±1.9	77.08±1.26	79.78±1.8	77.01±1.24	79.86±1.78	76.88±1.25	83.88±1.81	78.02±1.4	80.49±1.96	73.51±1.26^b	79.66±1.81^b	78.4±1.26	80.74±1.82
13	MFR	14.37±1.34	16.11±1.39	15.18±0.97	14.46±1.38	15.32±0.95	14.4±1.36	15.25±0.96	12.92±1.39	13.35±1.08	14.53±1.5	16.12±0.97	11.88±1.39	14.66±0.97	14.34±1.39
14	HFR	6.51±1.71	8.84±1.25	7.8±0.9	5.82±1.29	7.74±0.89	5.82±1.27	7.94±0.89	3.26±1.29	8.69±1.01	5.06±1.4	10.44±0.9^b	8.53±1.3^b	7.01±0.9	4.99±1.3
15	PLT	226.75±31.14	215.95±24.31	233.3±8.9	222.68±12.72	242.33±8.75	219±12.54	283±8.8^b	232.63±12.75^b	297.33±9.92^b	226.56±13.84^b	291.7±8.9^b	220.72±12.78^b	259.95±8.9	253.61±12.83
16	PDW	10.51±0.35	10.19±0.5	10.26±0.14	10.77±0.2	10.18±0.14	10.92±0.19	10.08±0.14^b	10.94±0.2	10.61±0.15	10.43±0.21	10.24±0.14	10.97±0.2	10.63±0.14	10.74±0.2
17	MPV	11.39±0.91	11.15±0.85	11.15±0.07	11.57±0.1	11.15±0.07^b	11.78±0.09	11±0.07^b	11.74±0.1	11.2±0.07	11.73±0.1	11.18±0.07	11.85±0.1^b	11.39±0.07	11.6±0.1
18	P-LCR	17.03±1.5	15.82±2.12	16.11±0.48	17.46±0.68	16.35±0.48	18.12±0.66	15.34±0.48	18.8±0.67	16.77±0.52	17.7±0.73	15.19±0.48^b	18.89±0.67	17.11±0.48	17.4±0.68
19	PCT	0.24±0.03	0.23±0.02	0.25±0.01	0.24±0.01	0.26±0.01	0.24±0.01	0.3±0.01	0.26±0.01	0.32±0.01	0.25±0.01	0.31±0.01	0.24±0.01	0.28±0.01	0.27±0.01
20	WBC	7.14±0.6	7.77±0.42	7.17±0.19	6.77±0.28	7.16±0.19	6.76±0.27	7.02±0.19	6.19±0.28	6.91±0.22	6.6±0.3	7.35±0.19	6.44±0.28	6.99±0.19	6.63±0.28
21	NEUTCount	3.83±0.33	4.49±0.32	3.84±0.18	3.56±0.25	3.82±0.17	3.57±0.25	3.86±0.17	2.97±0.25	3.61±0.2	3.3±0.28	3.99±0.18	3.22±0.25	3.6±0.18	3.61±0.26
22	LYMPCount	2.41±0.29	2.35±0.13	2.43±0.05	2.35±0.07	2.41±0.05	2.32±0.07	2.31±0.05	2.31±0.07	2.35±0.05	2.44±0.07	2.4±0.05	2.37±0.07	2.34±0.05	2.32±0.07
23	MONOCount	0.58±0.07	0.6±0.05	0.58±0.02	0.56±0.03	0.59±0.02	0.57±0.03	0.56±0.02	0.55±0.03	0.58±0.02	0.55±0.03	0.6±0.02	0.56±0.03	0.62±0.02	0.49±0.03
24	EOCount	0.28±0.06	0.3±0.05	0.29±0.02	0.27±0.03	0.3±0.02	0.26±0.03	0.25±0.02	0.33±0.03	0.32±0.02	0.28±0.03	0.31±0.02	0.25±0.03	0.38±0.02	0.18±0.03
25	BASOCount	0.02±0	0.02±0.01	0.02±0	0.02±0.01	0.02±0	0.02±0.01	0.02±0	0.02±0.01	0.03±0	0.01±0.01	0.02±0	0.03±0.01	0.02±0	0.02±0.01
26	NEUTPercentage	53.27±1.58	57.31±1.67	53.57±0.98	52.31±1.4	53.37±0.96	51.81±1.38	54.65±0.97	49.05±1.4	52.36±1.09	50.42±1.52	54.1±0.98	50.89±1.41	51.59±0.98	53.7±1.41
27	LYMPPercentage	34.22±2.06	30.82±1.55	33.92±0.82	34.79±1.18	33.83±0.81	34.99±1.16	33.49±0.81	36.13±1.18	34.07±0.92	36.41±1.28	32.95±0.82	36.11±1.18	33.32±0.82	35.75±1.19
28	MONOPer	8.42±1.35	7.74±0.53	8.36±0.24	8.42±0.35	8.44±0.24	8.88±0.34	8.14±0.24	9.24±0.35	8.44±0.27	8.8±0.38	8.4±0.24	8.82±0.35	9.31±0.24	7.61±0.35
29	EOPercentage	3.83±0.86	3.9±0.62	3.9±0.3	4.12±0.43	4.06±0.3	3.99±0.42	3.42±0.3	5.3±0.43	4.56±0.33	4.25±0.47	4.22±0.3	3.74±0.43	5.47±0.3	2.66±0.43
30	BASOPercentage	0.23±0.05	0.23±0.07	0.22±0.07	0.34±0.09	0.26±0.06	0.3±0.09	0.27±0.07	0.27±0.09	0.53±0.07	0.11±0.1	0.29±0.07	0.43±0.09	0.28±0.07	0.27±0.09
31	RET-He	23.75±0.42	23.39±0.27	23.76±0.16	23.99±0.22	23.87±0.15	24.26±0.22	23.59±0.15	24.24±0.22	24.23±0.17	24.09±0.24	26.15±0.16	24.35±0.22	26.05±0.16	24.55±0.23
32	RBC-He	23.73±0.2	23.93±0.19	23.84±0.08	23.69±0.11	23.65±0.08	24.05±0.11	23.59±0.08	23.76±0.11	24.24±0.09	23.54±0.12	26.03±0.08	23.83±0.11	26.28±0.08	23.93±0.11
