# Supplementary figures and images for: Long-term daily feeding of cannabidiol is well-tolerated by healthy dogs
Source: Front Vet Sci. 2022 Sep 21;9:977457. doi: 10.3389/fvets.2022.977457 (PMC9533147; doi:10.3389/fvets.2022.977457)

(A)

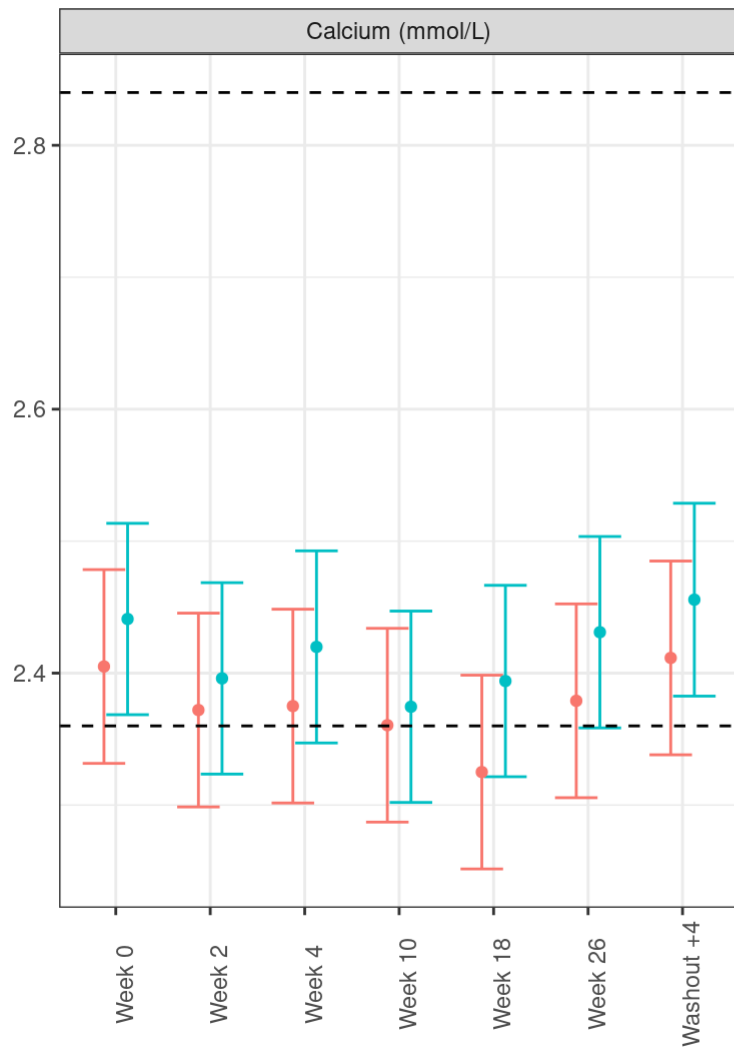

(B)

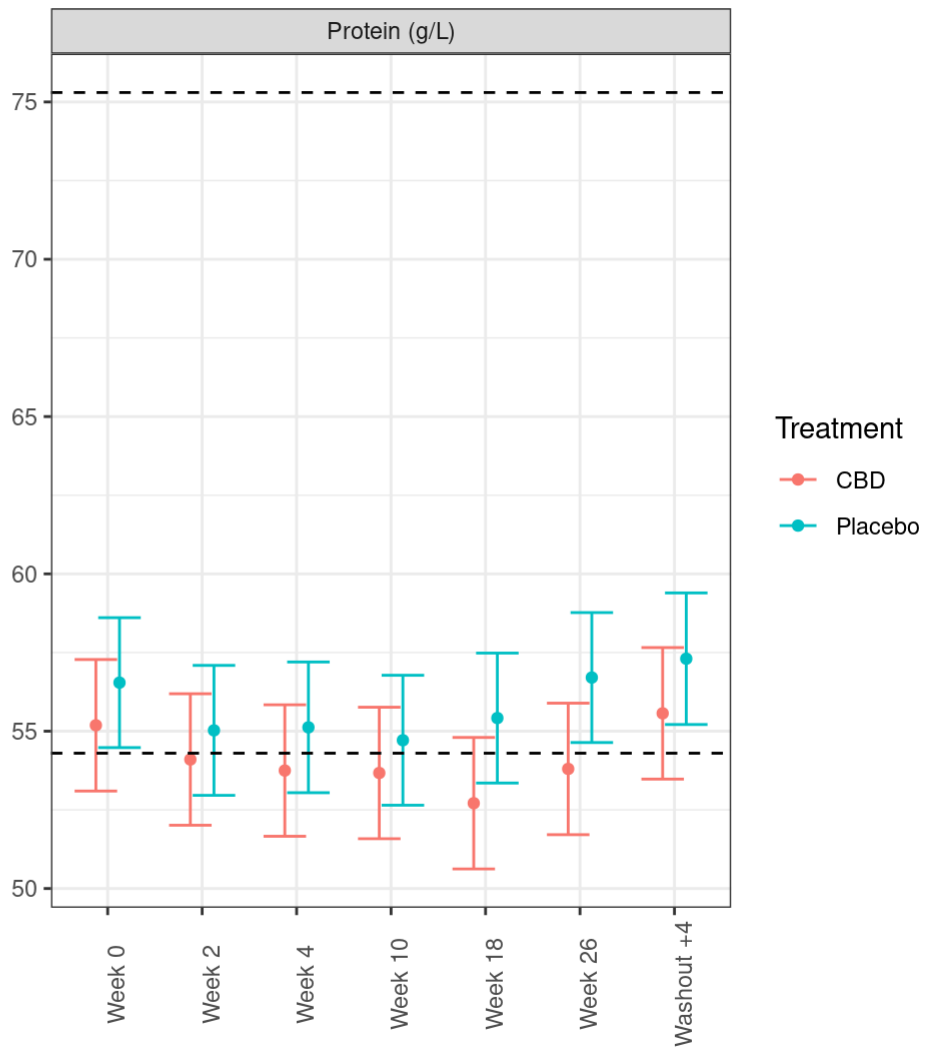

Supplement: Supplementary Figure 1 (S2) — Plasma concentrations (mean and 95% confidence intervals) of calcium [(A); mmol/L] and protein [(B); g/L] in dogs dosed with CBD (red) and placebo (blue). Week 0 depicts the baseline measure before daily oral dosing of CBD/placebo. Dotted line depicts upper and lower reference ranges as specified by IDEXX Laboratories. [file Data_Sheet_2.PDF]

# CBD

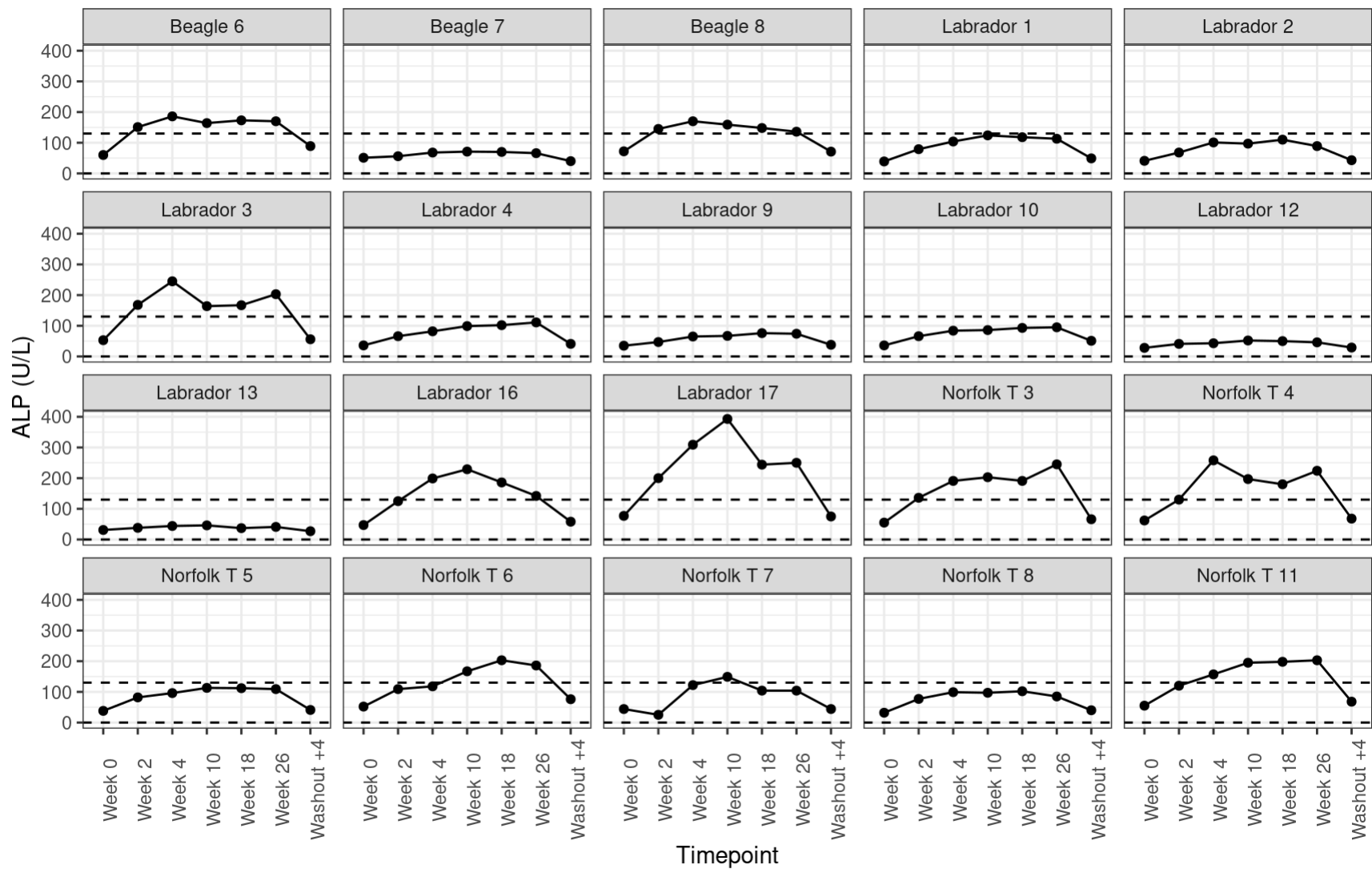

Supplement: Supplementary Figure 2 (S3) — Plasma activity levels of total alkaline phosphatase (ALP; U/L) at each study time-point, in each of 20 individual dogs receiving CBD. Breed of each dog is detailed above each individual graph. Dotted line depicts upper and lower reference ranges as specified by IDEXX Laboratories. [file Data_Sheet_3.PDF]

Plasma CBD concentration (ng/ml)

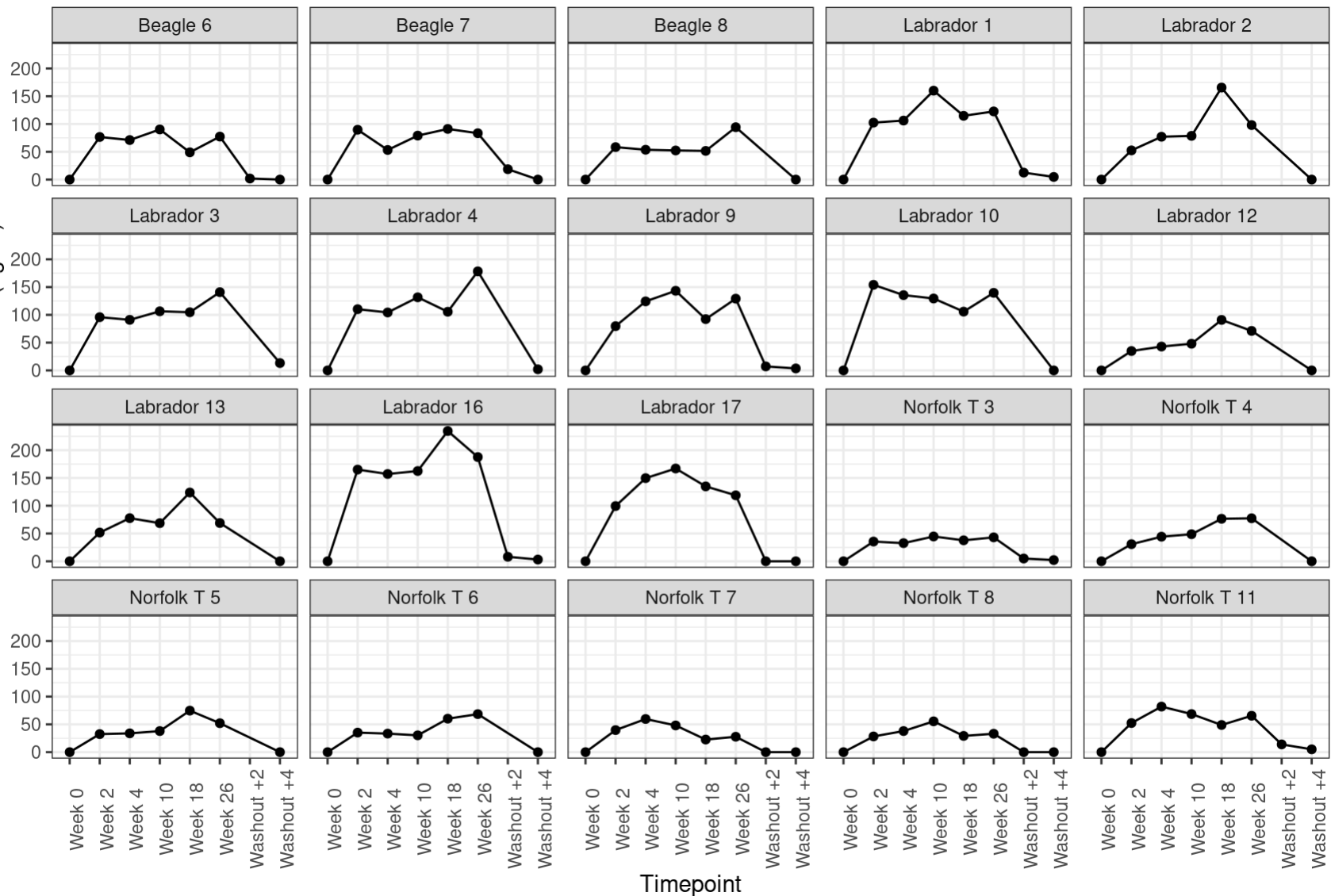

Supplement: Supplementary Figure 3 (S5) — Plasma levels of CBD (ng/ml) at each study time-point, in each individual dog receiving CBD. Breed of each dog is detailed in the legend above each graph. Week 0 depicts the baseline. [file Data_Sheet_5.PDF]

## CBD concentration in faeces

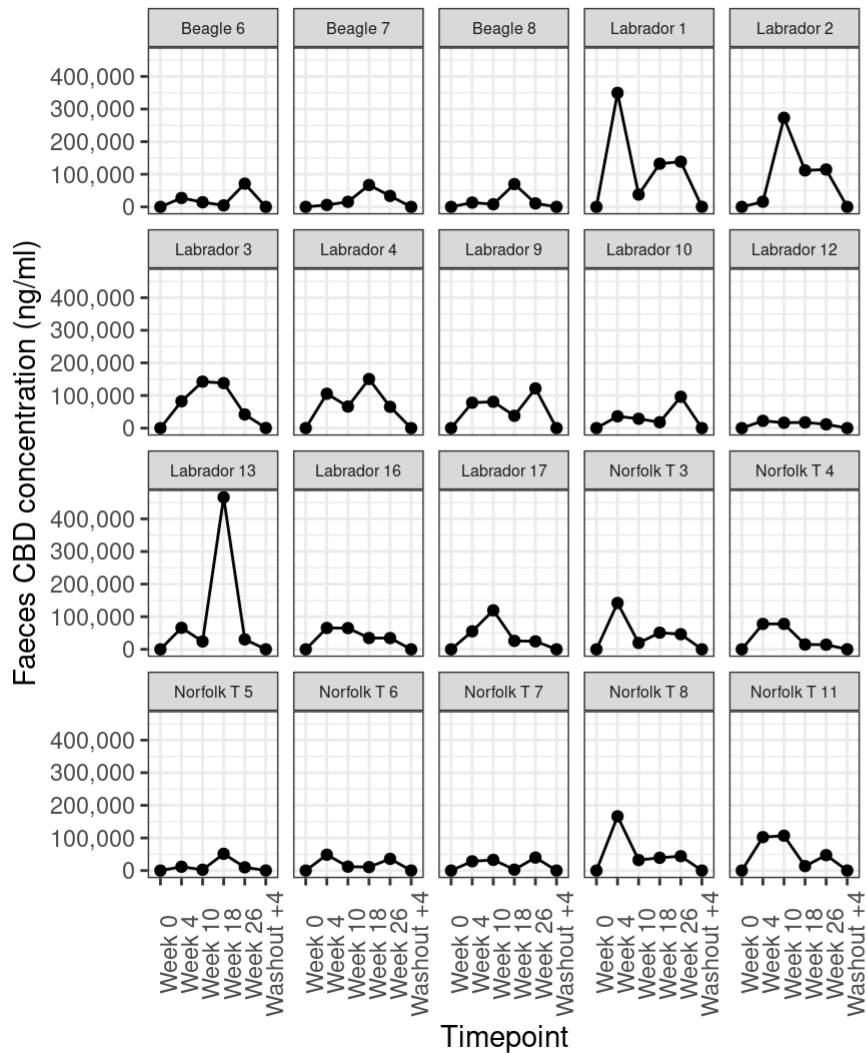

## CBD concentration in urine

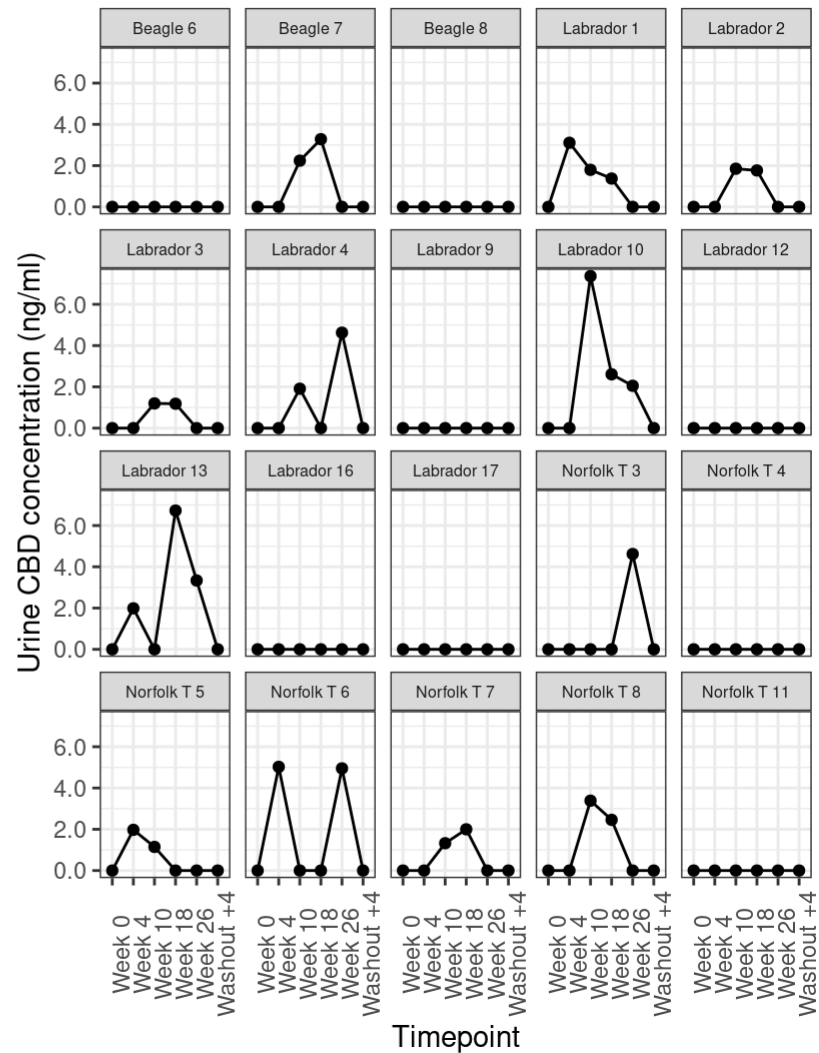

Supplement: Supplementary Figure 4 (S6) — CBD concentrations in feces [(A); ng/g] and urine [(B); ng/ml] for each CBD-dosed dog at each study time-point. Week 0 depicts the baseline. [file Data_Sheet_6.PDF]
