# Supplementary material for: Long-term daily feeding of cannabidiol is well-tolerated by healthy dogs
Source: Front Vet Sci. 2022 Sep 21;9:977457. doi: 10.3389/fvets.2022.977457 (PMC9533147; doi:10.3389/fvets.2022.977457)
Supplement: Supplementary Table 2 (S4) — Full suite of biochemical parameters measured in the plasma at each time point for both CBD- and placebo- fed dogs. Bold reflects significance within the treatment group at that time point and b indicates significance between the treatment groups at that time-point. [file Data_Sheet_4.PDF]

	Parameters	Week0CBD	Week0Placebo	Week2CBD	Week2Placebo	Week4CBD	Week4Placebo	Week10CBD	Week10Placebo	Week18CBD	Week18Placebo	Week26CBD	Week26Placebo	Wash4CBD	Wash4Placebo
1	ALT	35.96±3.76	33.64±2.52	35.41±1.9	35.7±2.68	34.74±1.9	37.1±2.7	35.71±1.9	36.36±2.68	36.82±1.9	35.71±2.68	37.02±1.9	34.43±2.68	37.7±1.9	33.77±2.73
2	Protein	55.19±0.78	56.54±0.86	54.1±0.49	54.76±0.69[b]	53.75±0.49[b]	55.21±0.69	53.67±0.49[b]	54.87±0.69[b]	**52.71±0.49**[b]	**56.54±0.69**	**53.8±0.49**	**56.74±0.69**	55.57±0.49	55.57±0.7
3	Albumin	28.66±0.51	28.97±0.57	28.17±0.25	28.9±0.35	27.61±0.25[b]	29.4±0.36	27.4±0.25[b]	29.34±0.35	**26.61±0.25**[b]	**30.37±0.35**	**26.81±0.25**[b]	**30.42±0.35**	27.86±0.25	29.73±0.36
4	Glucose	5.65±0.14	5.46±0.12	5.66±0.08	5.75±0.11	5.65±0.08	5.72±0.11	5.77±0.08	5.73±0.11	5.57±0.08	5.76±0.11	5.64±0.08	5.83±0.11	5.61±0.08	5.98±0.11
5	InorgPhos	1.22±0.04	1.3±0.05	1.23±0.04	1.19±0.05	1.2±0.04	1.24±0.05	1.21±0.04	1.13±0.05[b]	1.18±0.04	1.27±0.05	1.21±0.04	1.19±0.05	1.24±0.04	1.21±0.05
6	AST	32.4±2.59	28.25±1.95	31.23±1.07	34.28±1.51	31.29±1.07	32.6±1.53	32.49±1.07	33.91±1.51	30.15±1.07	34.4±1.51	30.13±1.07	34.27±1.51	32.08±1.07	32.96±1.54
7	Calcium	2.41±0.03	2.44±0.03	2.37±0.01	2.39±0.02[b]	2.38±0.01	2.41±0.02	2.36±0.01[b]	2.38±0.02[b]	2.33±0.01[b]	2.44±0.02[b]	2.38±0.01	2.42±0.02	2.41±0.01	2.41±0.02
8	Cholsterol	4.11±0.28	4.52±0.27	4.02±0.1	4.25±0.14	4.12±0.1	4.25±0.14	4.19±0.1	4.15±0.14	4.13±0.1	4.32±0.14	4.22±0.1	4.38±0.14[b]	**4.18±0.1**	**4.49±0.14**
9	Urea	6.06±0.22	5.63±0.3	6.12±0.17	6±0.24	6±0.17	6.27±0.24	5.97±0.17	6.28±0.24	6.14±0.17	6.09±0.24	5.99±0.17	6.36±0.24	6±0.17	6.18±0.25
10	Triglycerides	0.62±0.06	0.58±0.05	0.62±0.03	0.62±0.05	0.67±0.03	0.55±0.05	0.59±0.03	0.63±0.05	0.59±0.03	0.64±0.05	0.59±0.03	0.62±0.05	0.62±0.03	0.62±0.05
11	AlkPhos	48.69±10.77	56.61±13.75	97.95±7.51[b]	3.34±10.61	**138.54±7.51**[b]	**-34.97±10.69**	**145.09±7.51**[b]	**-48.66±10.61**	**134.69±7.51**[b]	**-36.91±10.61**	**136.09±7.51**[b]	**-38.11±10.61**	54.99±7.51	42.21±10.78
12	Creatinine	75.25±7.1	72.9±3.01	74.13±1.65	75.26±2.33	74.44±1.65	76.8±2.35	75.44±1.65	76.21±2.33	77.03±1.65	75.82±2.33	78.08±1.65	75.88±2.33	73.88±1.65	82.12±2.37
13	Sodium	146.41±0.5	146.41±0.41	145.61±0.3	146.12±0.43[b]	145.07±0.3[b]	146.88±0.43	144.44±0.3[b]	146.74±0.43[b]	145.12±0.3[b]	146.48±0.43[b]	144.86±0.3[b]	146.48±0.43[b]	145.63±0.3	146.6±0.43
14	Potassium	4.19±0.06	4.13±0.08	4.12±0.07	4.32±0.09	4.22±0.07	4.21±0.1	4.17±0.07	4.21±0.09	4.23±0.07	4.21±0.09	4.17±0.07	4.37±0.09	4.3±0.07	4.12±0.1
15	Chloride	114.6±0.41	114.9±0.58	113.76±0.36	114.32±0.51[b]	113.87±0.36	114.09±0.51[b]	113.26±0.36[b]	115.05±0.51	114.7±0.36	113.42±0.51[b]	114.02±0.36	114.34±0.51	113.94±0.36	114.44±0.51
16	Globulin	26.52±0.51	27.57±0.57	25.93±0.34	25.85±0.48[b]	26.13±0.34	25.8±0.48[b]	26.26±0.34	25.53±0.48[b]	26.09±0.34	26.16±0.48	26.98±0.34	26.31±0.48	27.7±0.34	25.83±0.49
17	AGRatio	1.08±0.02	1.05±0.03	1.09±0.01	1.12±0.02[b]	1.06±0.01	1.14±0.02	1.05±0.01	1.15±0.02	1.02±0.01[b]	1.16±0.02	1±0.01[b]	1.16±0.02	1.01±0.01	1.15±0.02
18	SodiumOverPotassium	35.02±0.49	35.59±0.69	35.41±0.53	33.84±0.74	34.56±0.53	34.93±0.75	34.76±0.53	34.93±0.74	34.37±0.53	34.84±0.74	34.83±0.53	33.72±0.74	33.96±0.53	35.62±0.75
19	GGT	3.33±0.54	3.64±0.33	3.56±0.29	2.45±0.42	3.8±0.29	2.69±0.42	3.59±0.29	2.63±0.42	3.41±0.29	2.6±0.42	3.31±0.29	2.16±0.42[b]	3±0.29	2.75±0.42
20	Bilirubin	2.67±0.32	2.9±0.26	2.3±0.24	2.62±0.34	2.49±0.24	2.51±0.34	2.11±0.24	2.74±0.34	2.56±0.24	2.53±0.34	2.57±0.24	2.06±0.34	2.72±0.24	2.28±0.35
21	BileAcids	2.03±1.54	3.68±1.84	2.03±1.66	4.53±2.34	2.65±1.66	0.7±2.36	1.97±1.66	2.19±2.34	1.17±1.66	4.83±2.34	1.99±1.66	1.53±2.36	3.02±1.66	5.49±2.38
